# Supplementary material for: Schizophrenia-associated NRXN1 deletions induce developmental-timing- and cell-type-specific vulnerabilities in human brain organoids
Source: Nat Commun. 2023 Jun 24;14:3770. doi: 10.1038/s41467-023-39420-6 (PMC10290702; doi:10.1038/s41467-023-39420-6)
Supplement: Supplementary file 1 — Supplementary Information [file 41467_2023_39420_MOESM1_ESM.pdf]

Schizophrenia-associated *NRXN1* deletions induce developmental timing- and cell-type-specific vulnerabilities in human brain organoids

Supplementary information

Supplementary Figures

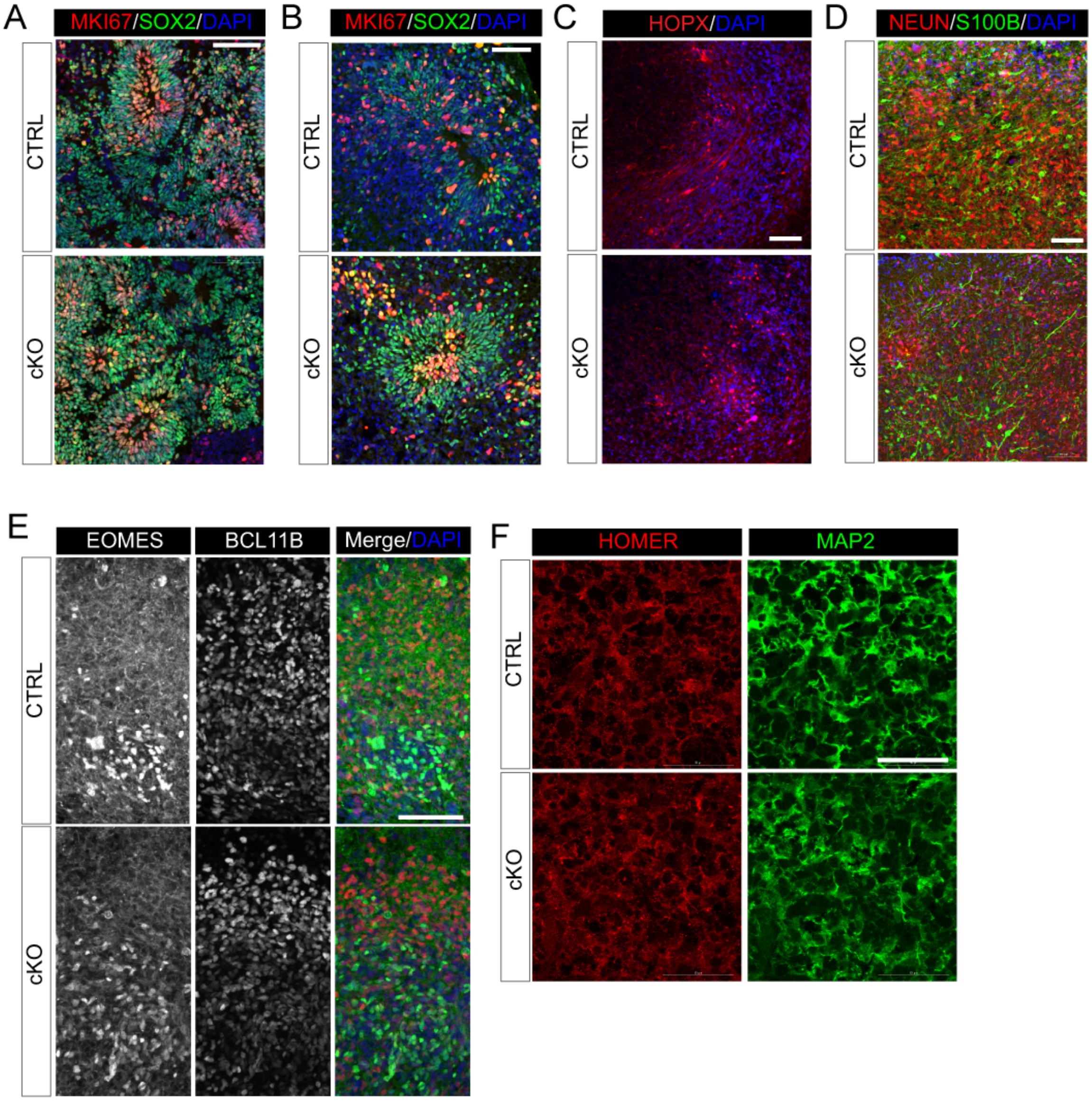

Figure S1. *NRXN1* cKO engineered forebrain organoids undergo key developmental processes.

Representative confocal images of brain organoid sections immunostained with antibodies against key markers across time points – day 21 (A; scale bar 100 μm), day 50 (B; scale bar 50 μm), day 100 (C-E; scale bars 100 μm), and day 150 (F; scale bar 50 μm). MIK67, proliferative cells; SOX2, ventricular zones; HOPX, oRGs; NEUN, pan-neuronal marker; S100B, astrocytes; EOMES, intermediate progenitors; BCL11B, early born neurons; HOMER, post-synaptic marker; MAP2, dendritic marker.

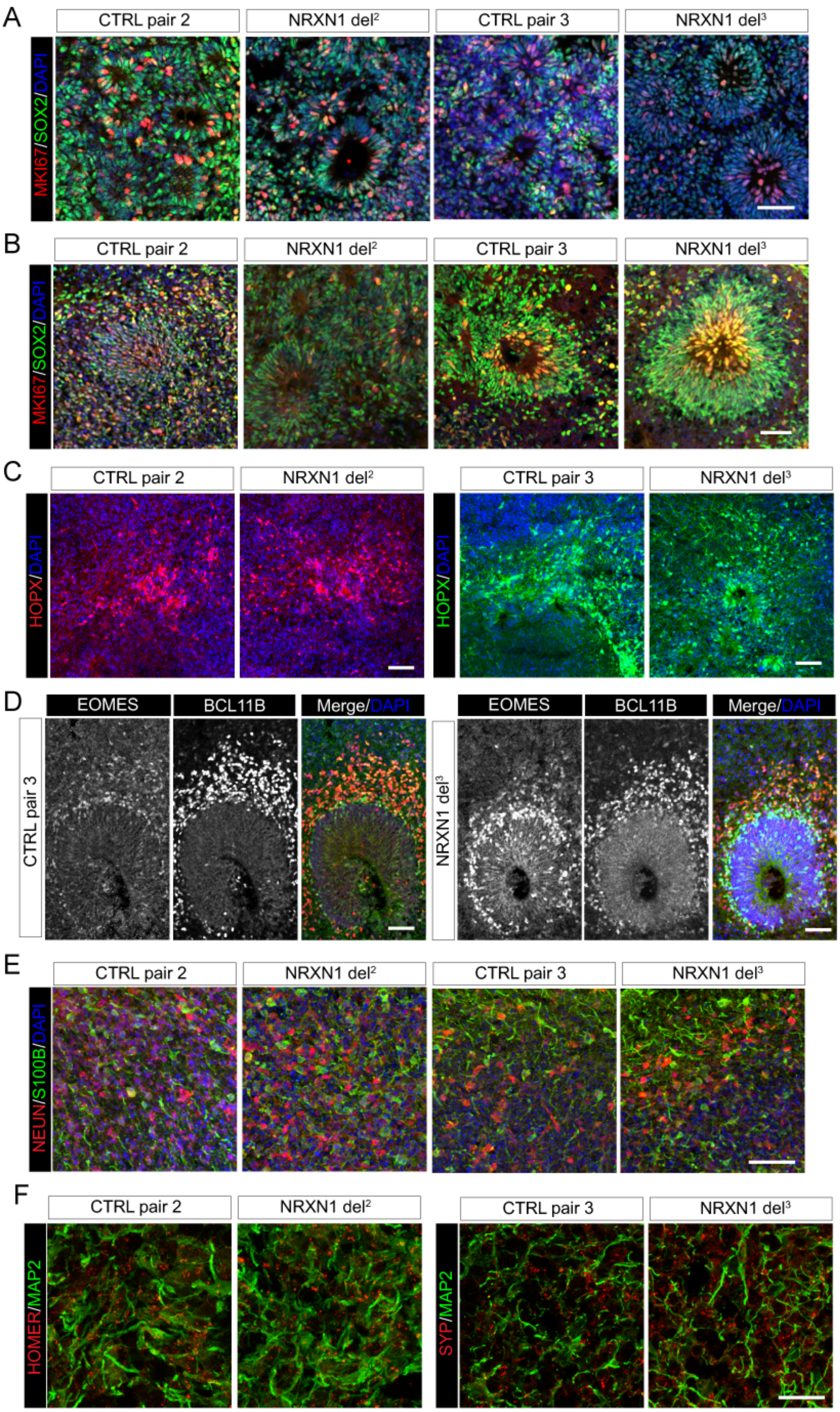

A
CTRL pair 2
NRXN1 del2
CTRL pair 3
NRXN1 del3
MKI67/SOX2/DAPI
B
CTRL pair 2
NRXN1 del2
CTRL pair 3
NRXN1 del3
MKI67/SOX2/DAPI
C
CTRL pair 2
NRXN1 del2
CTRL pair 3
NRXN1 del3
HOPX/DAPI
HOPX/DAPI
D
EOMES
BCL11B
Merge/DAPI
EOMES
BCL11B
Merge/DAPI
CTRL pair 3
NRXN1 del3
E
CTRL pair 2
NRXN1 del2
CTRL pair 3
NRXN1 del3
NEUN/S100B/DAPI
F
CTRL pair 2
NRXN1 del2
CTRL pair 3
NRXN1 del3
HOMER/MAP2
SYP/MAP2

Figure S2. SCZ-*NRXN1*^{del} and control donor derived forebrain organoids undergo key developmental processes.

Representative confocal images of brain organoid sections immunostained with antibodies against key markers across time points – day 21 (A; scale bar 50 μm), day 50 (B; scale bar 50 μm), day 100 (C; scale bar 100 μm, D; scale bar 50 μm, E-F; scale bars 25 μm). MIK67, proliferative cells; SOX2, ventricular zones; HOPX, oRGs; NEUN, pan-neuronal marker; S100B, astrocytes; EOMES, intermediate progenitors; BCL11B, early born neurons; HOMER, post-synaptic marker; SYP, pre-synaptic marker; MAP2, dendritic marker.

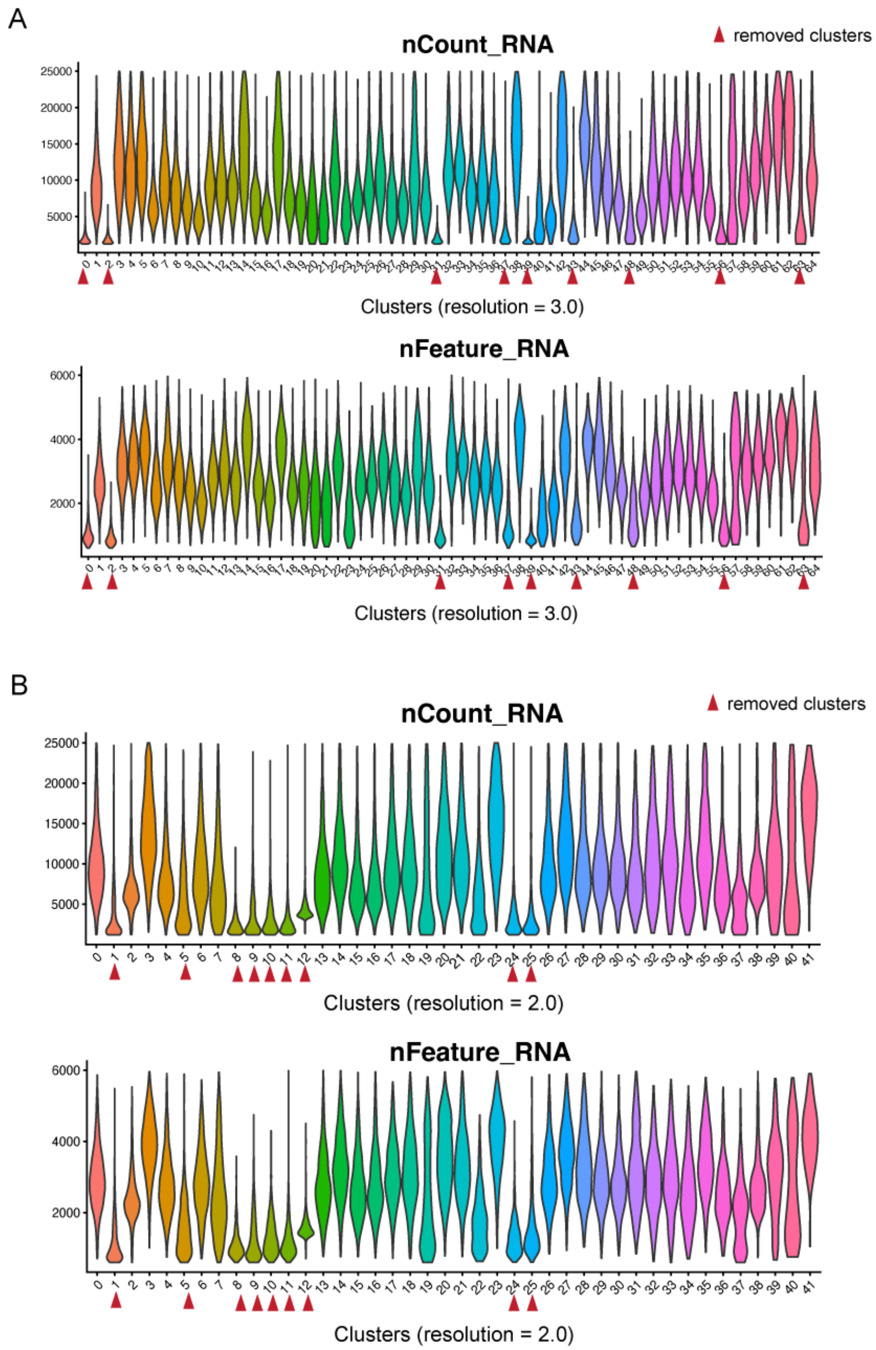

Figure S3. Quality control of scRNAseq data obtained from donor-derived and engineered brain organoids.

(A) Violin plots showing distribution of the number of Unique Molecular Identifier (UMI) counts per cell (top) and the number of unique genes per cell (bottom) of Louvain clusters (resolution = 3.0) from the integrated donor-derived organoid scRNAseq data from batch 1. Clusters with low levels of sequencing depth indicated by red arrows were removed from the final analysis. (B) Similar to (A), low-sequencing-depth clusters (resolution = 2.0) from the integrated engineered brain organoid scRNAseq data were removed.

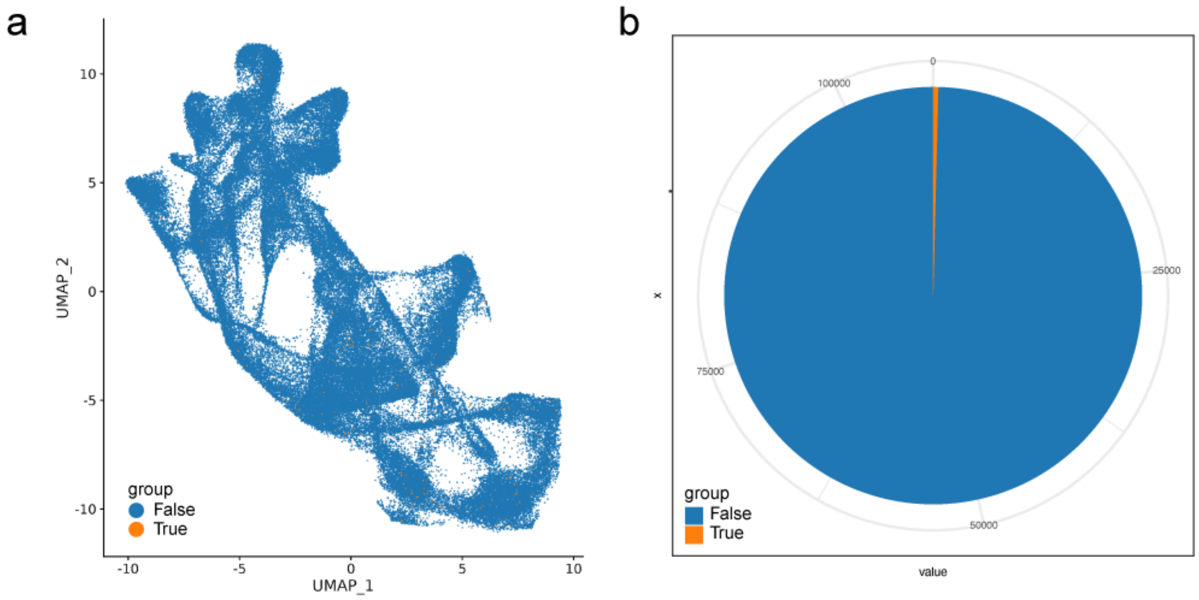

Figure S4. Doublet detection in the single cell data.
(a) Doublet detection was conducted using Scrublet in the integrated single cell data from both donor-derived and engineered organoids (See methods). Orange dots (True) and blue dots (False) represent doublets and single cells, respectively. (b) The count of doublets (True) is shown in a pie chart, representing a low ratio of doublets compared with single cells.

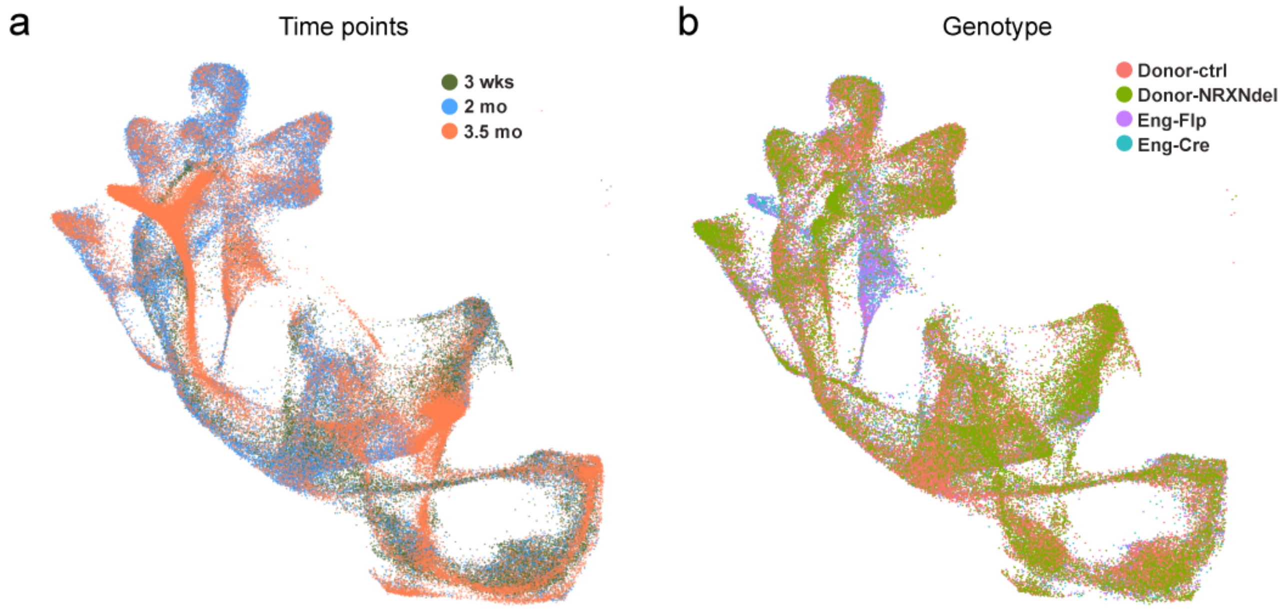

Figure S5. UMAPs separated by time points and genotypes.
UMAPs showing distributions of time points (a) and genotypes (b) of developing brain organoid samples used for scRNAseq. A total of 10 samples were processed for the engineered deletion (Cre) control (Flp) – n=1 for 3 wk Cre and Flp; n=2 for 2 mo Cre and Flp; n=2 for 3.5 mo Cre and Flp. A total of 16 samples were processed – 2 SCZ-*NRXN1*^{del} donors and 2 Ctrl donors for 3 wk; 2 SCZ-*NRXN1*^{del} donors and 2 Ctrl donors for 2 mo; 4 SCZ-*NRXN1*^{del} donors and 4 Ctrl donors for 3.5 mo. In total, 156,966 single cell transcriptomes are projected on these UMAPs and processed for analyses. See Table S1 for details on samples.

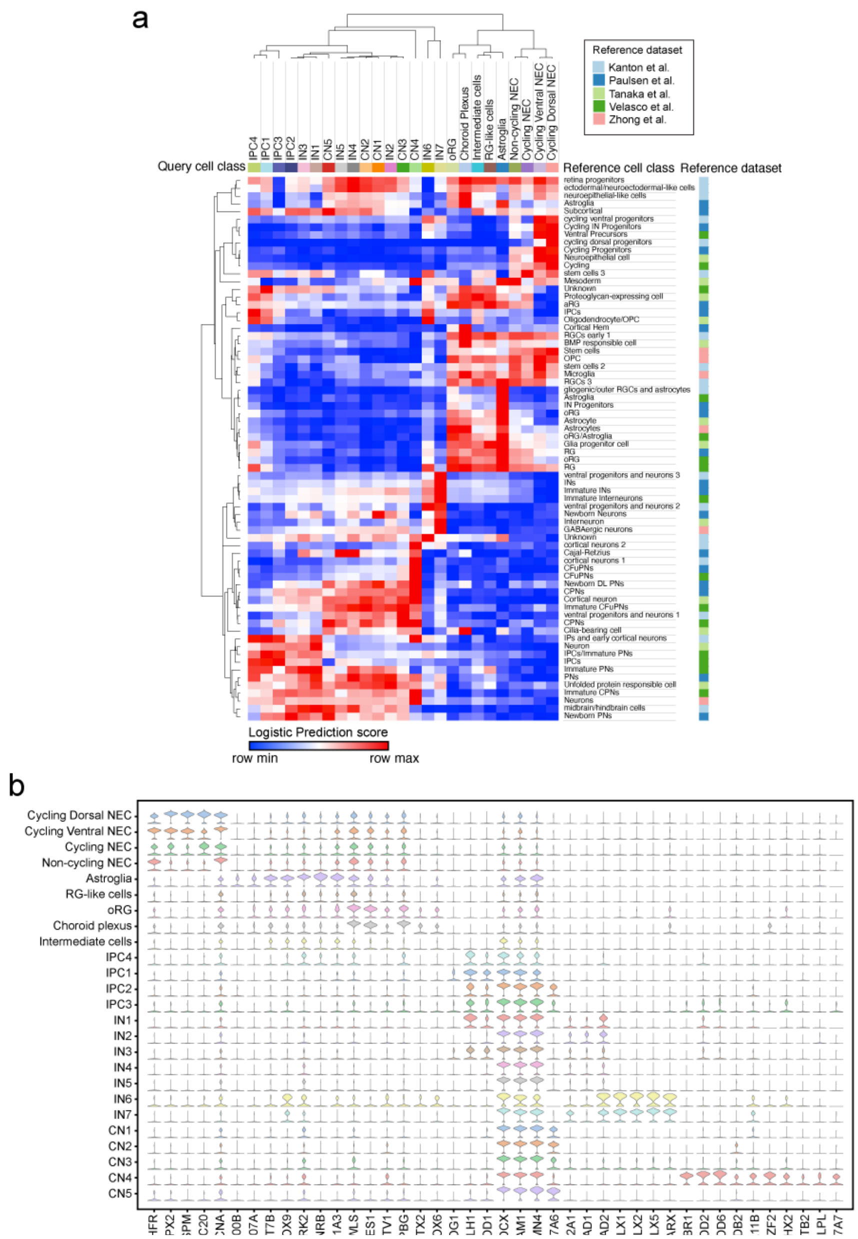

Figure S6. Stacked violin plots showing key molecular markers for each cell class.

(a) Similarity of cell classes in the current study and reference datasets measured by logistic regression models. Classification models were trained for each cell class in the published four brain organoid single-cell datasets and one fetal brain classification (see Methods). Average prediction scores were calculated for cells in each query cell class. Average prediction scores were shown in the heatmap to show the similarity of cell classes between our dataset and reference brain organoid and fetal brain datasets (see Methods). (b) Normalized expression level of each marker gene is shown for each cell class in the integrated single-cell data.

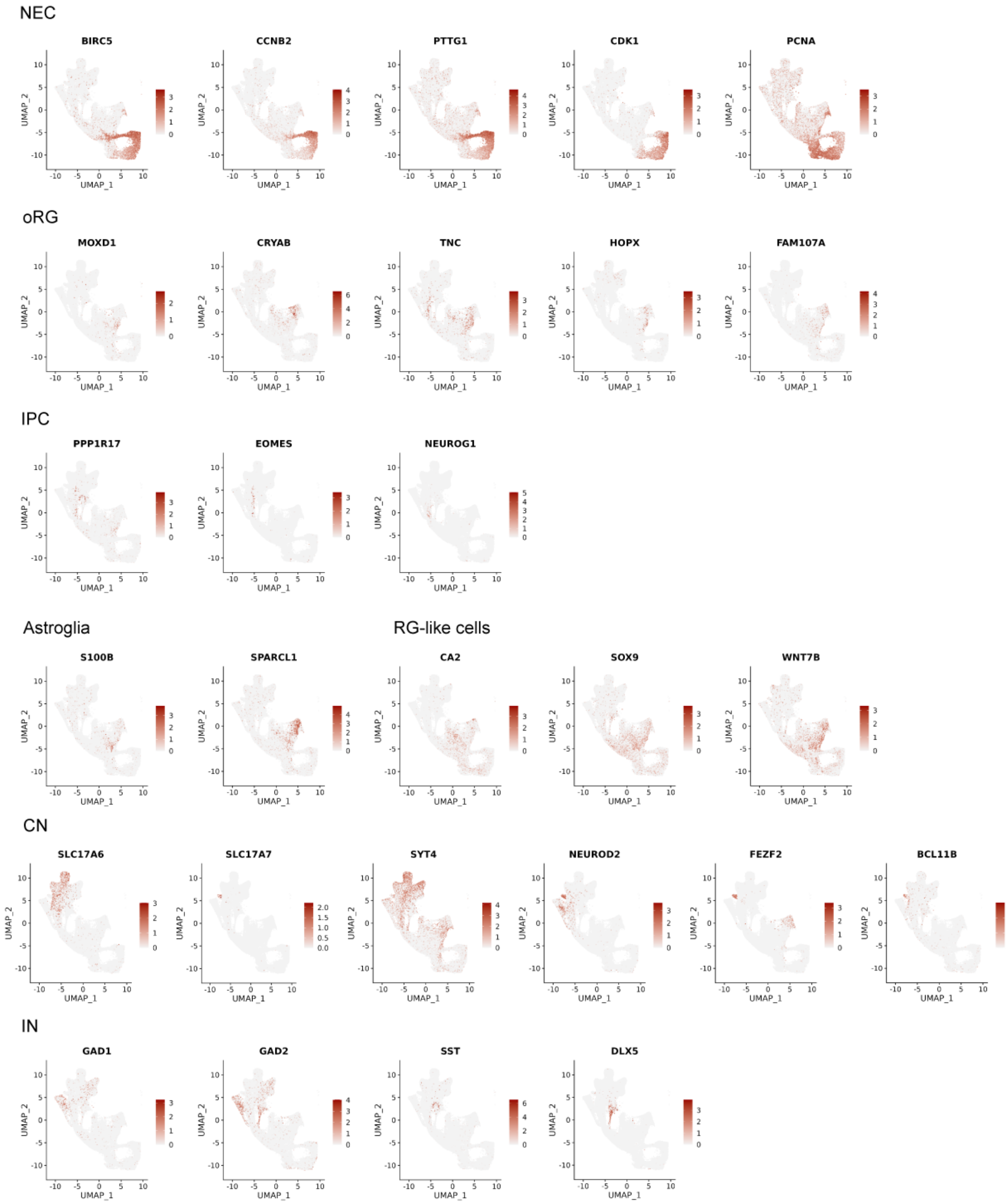

Figure S7. Distribution of key markers on UMAP.

UMAPs showing the distributions of normalized expression levels of marker genes in various cell classes, including neural precursor cells (NECs), outer radial glial cells (oRG), intermediate precursor cells (IPC), astroglia cells, cortical glutamatergic excitatory neurons (CN), and cortical GABAergic inhibitory neurons (IN).

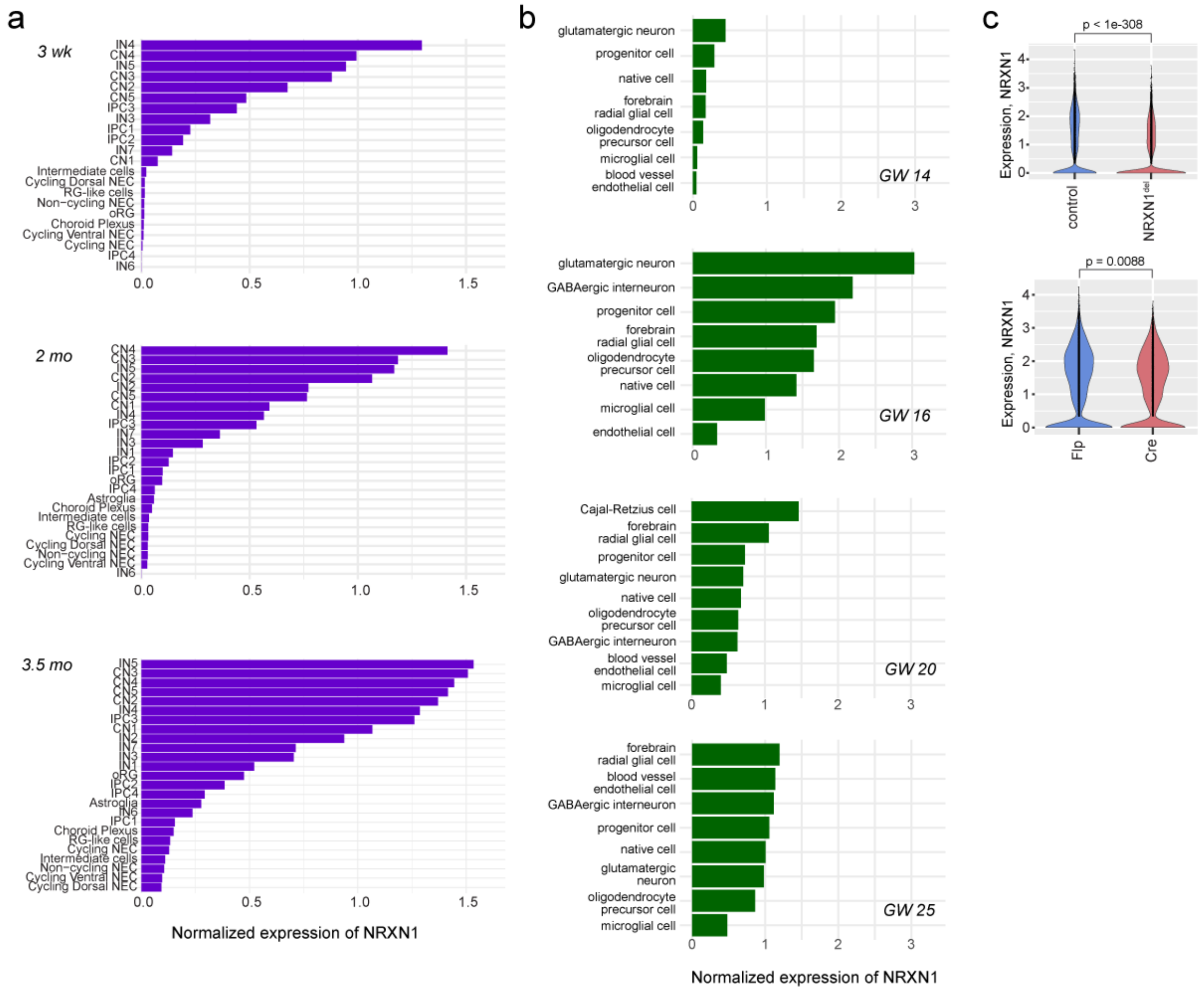

Figure S8. Single cell expression of *NRXN1* transcripts in the developing forebrain organoids and human fetal cortex.

Bar graphs showing normalized single cell expression of *NRXN1* mRNAs in the developing forebrain organoids derived from control donors used in our study (a) and the human fetal tissue from 14, 16, 20, and 25 GWs (b). Postmortem data was mined from the human neocortex single-cell transcriptome study (14-25 GWs; Badhuri et al., 2021). Normalized values “Log(CP10K+1) (Seurat Default Normalization)” were calculated as the NRXN1 expression levels. (c) Violin plots for all cells in both genotype groups in 3.5 mo organoids. Wilcoxon test p-values are noted.Top- donor derived organoids; bottom- engineered organoids.

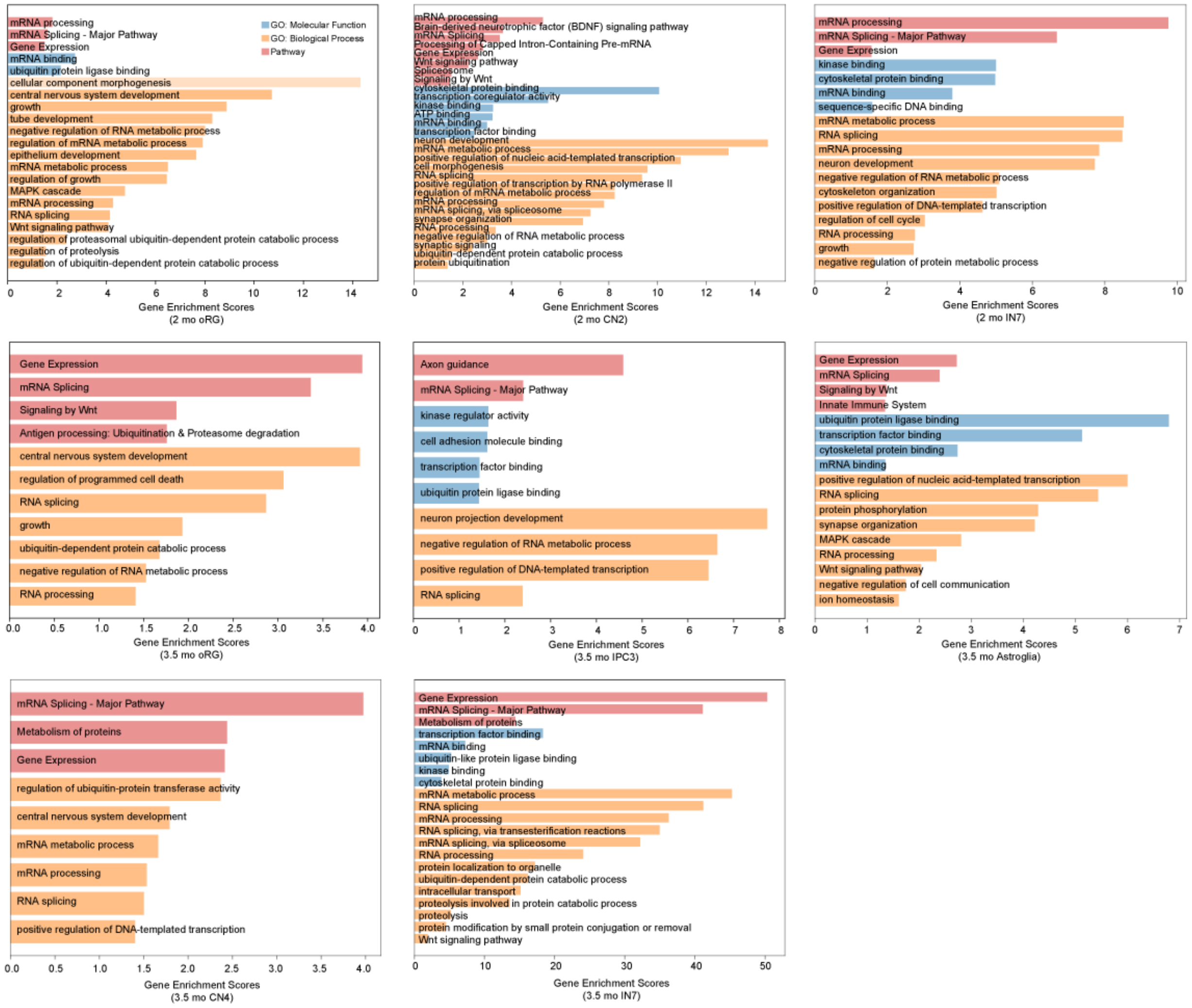

Figure S9. Gene set enrichment analysis of DEGs from *NRXN1* cKO brain organoids. Representative enriched gene sets shown for GSEA results of DEGs of multiple cell classes using ToppGene. Different categories of Gene Ontology are shown in different colors (blue: molecular function, orange: biological process, and red: pathway). Gene Set Enrichment Analysis (GSEA) was employed and enrichment scores were defined as $-log_{10}(FDR\ adjusted\ p\ values)$ to represent the associations between DEG sets and Gene Ontology gene sets.

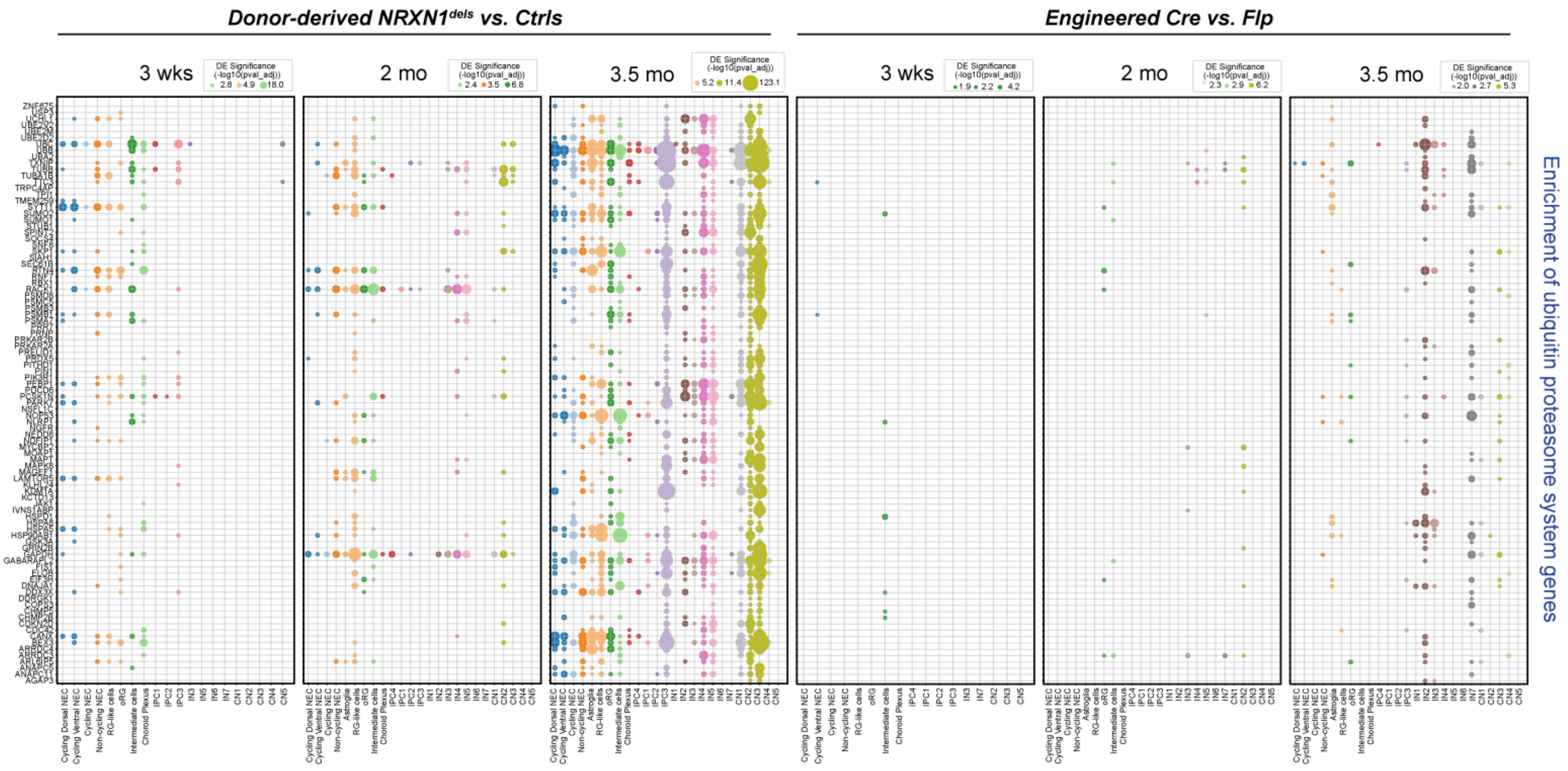

Figure S10. Differential enrichment of UPS biology DEGs across time points and cell types.
Significance of differential expression of selected UPS genes across time points and cell types in both donor-derived brain organoids (left) and engineered brain organoids (right). The size of each dot represents the level of DE significance of each gene in each cell class. Two-sided Wilcoxon rank-sum tests were employed for the DE tests, and DE significance was defined as $-log_{10}(FDR\ adjusted\ p\ values)$.

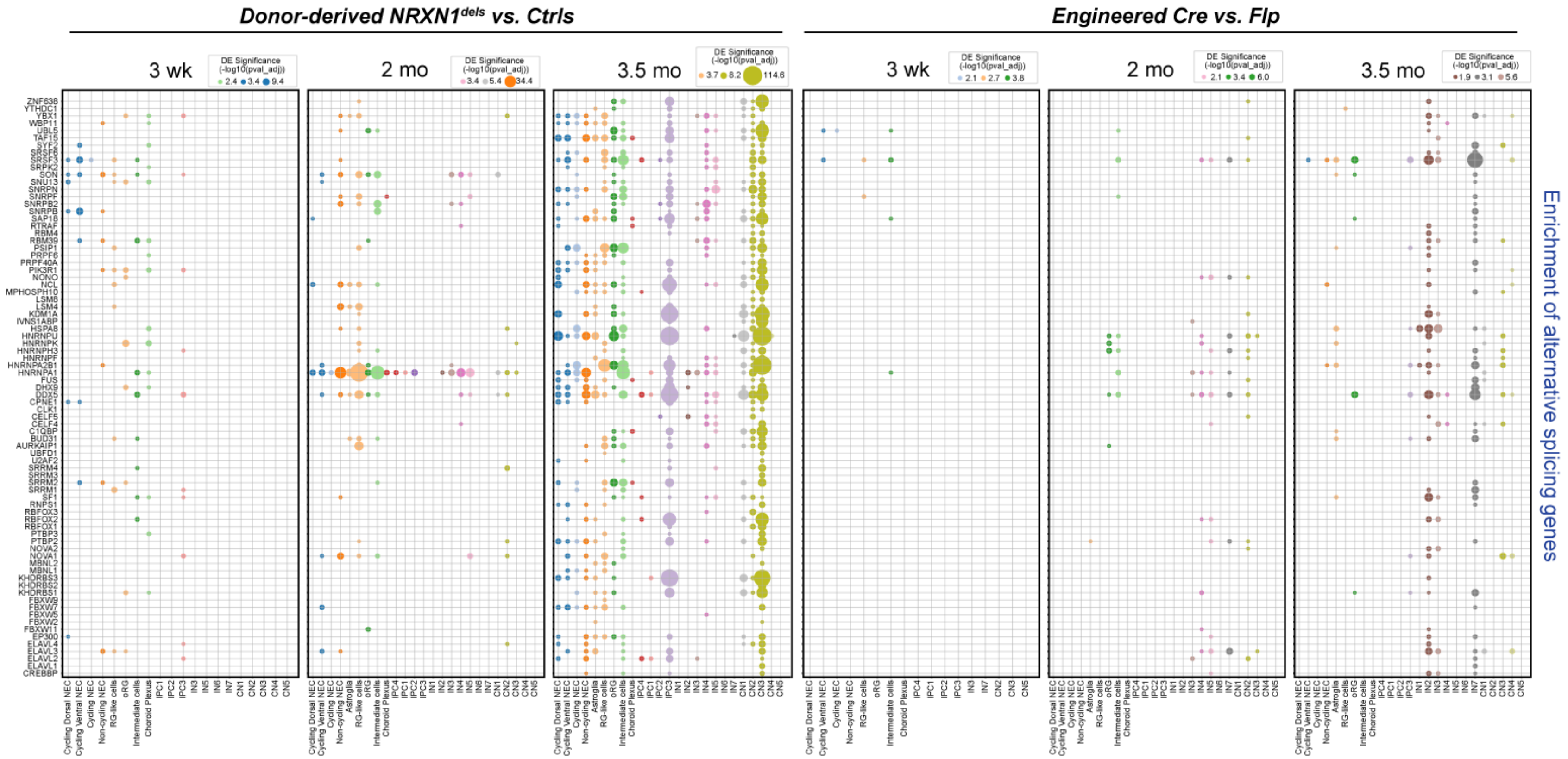

Figure S11. Differential enrichment of alternative splicing regulator DEGs across time points and cell types. Significance of differential expression of selected genes important for alternative splicing across time points and cell types in both donor-derived brain organoids (left) and engineered brain organoids (right). The size of each dot represents the level of DE significance of each gene in each cell class. Two-sided Wilcoxon rank-sum tests were employed for the DE tests, and DE significance was defined as $-log_{10}(FDR\ adjusted\ p\ values)$.

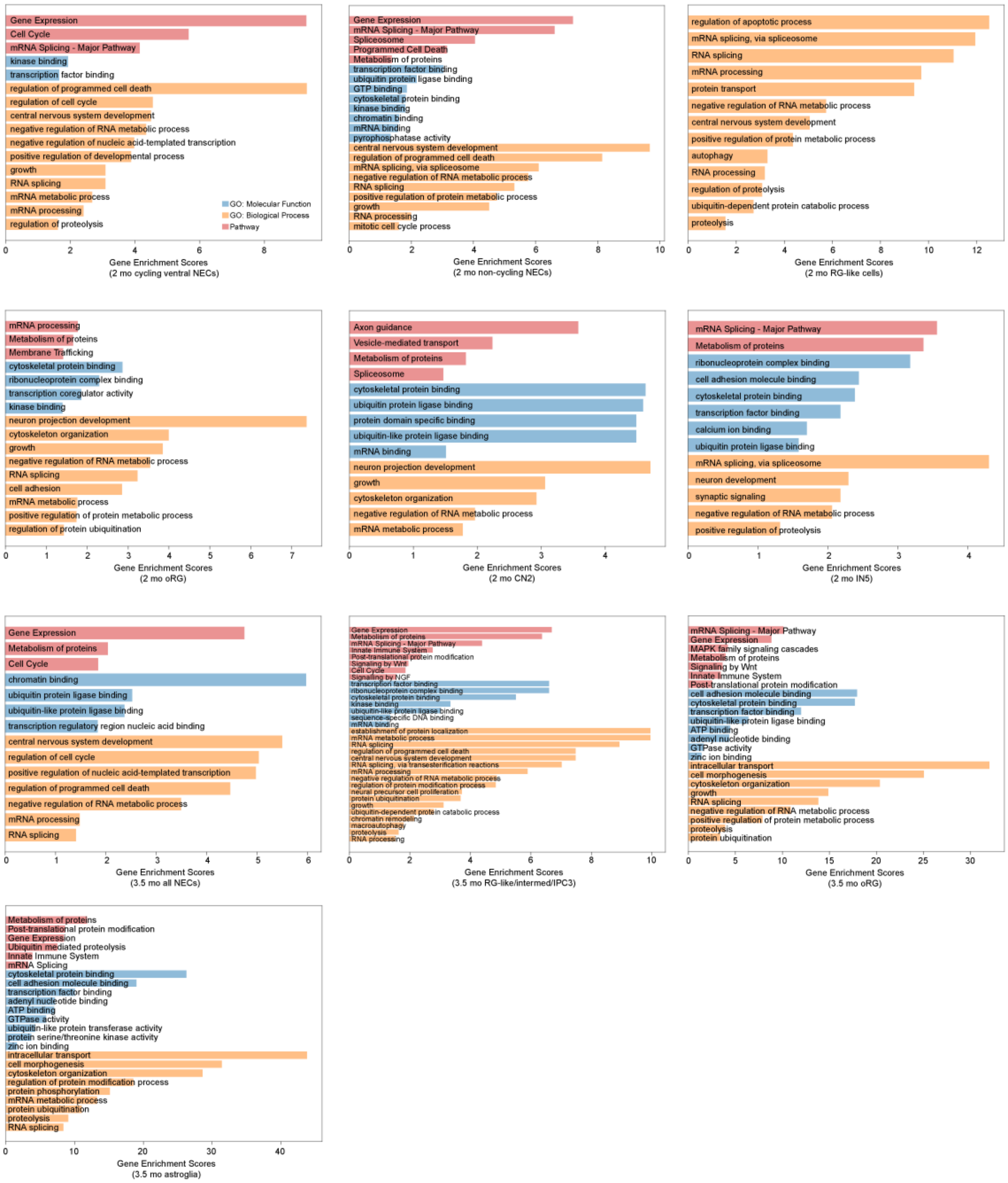

Figure S12. Gene set enrichment analysis of DEGs from SCZ-*NRXN1* deletion donor brain organoids.

Representative enriched gene sets shown for GSEA results of DEGs of multiple cell classes using ToppGene. Different categories of Gene Ontology are shown in different colors (blue: molecular function, orange: biological process, and red: pathway). Gene Set Enrichment Analysis (GSEA) was employed and enrichment scores were defined as $-log_{10}(FDR\ adjusted\ p\ values)$ to represent the associations between DEG sets and Gene Ontology gene sets.

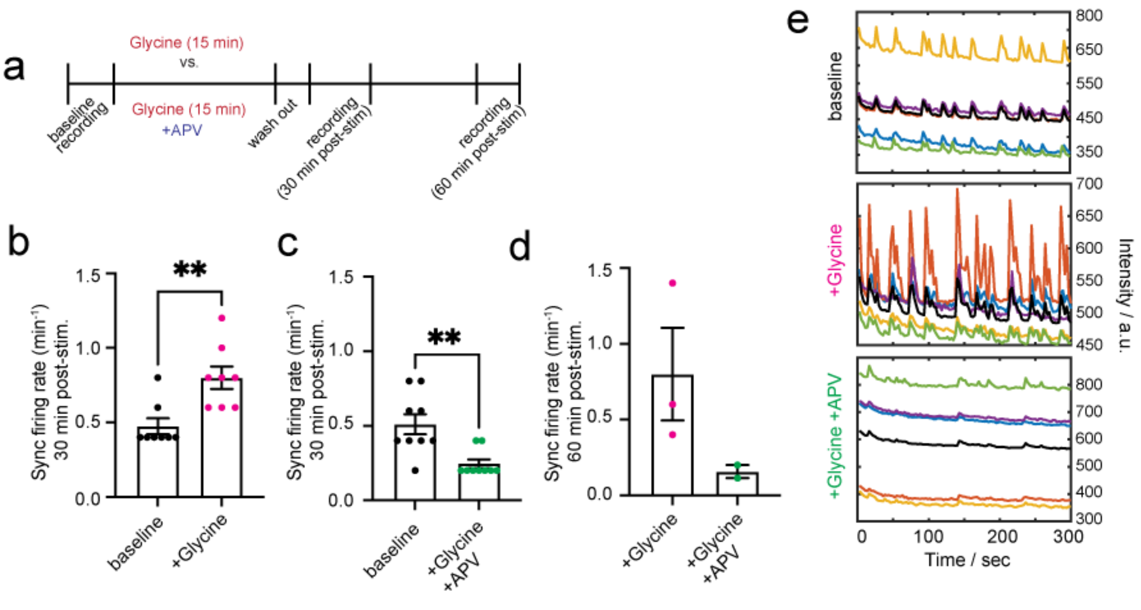

Figure S13. Glycine stimulation induces sustained increase in neuronal activity that is sensitive to NMDAR blockade.

(a) Schematic illustrating Ca^{2+} imaging workflow for glycine stimulation in live brain organoids expressing soma-GCaMP6fs. (b) Average synchronous firing rate before (baseline) and after glycine stimulation (+Glycine). Measurements were taken 30 minutes post-stimulation. N=8. (c) Average synchronous firing rate before (baseline) and after glycine stimulation with NMDAR blocker APV (+Glycine, +APV). Measurements were taken 30 minutes post-stimulation. N=9. (d) Potentiated response is sustained after 60 minutes post-stimulation (+Glycine) which is inhibited in the presence of APV (+Glycine, +APV). N=2. (e) Representative traces showing raw intensity traces with averaged intensities plotted in bolded black for each condition. Each data point represents averaged data from a single field of view (FOV) consisting of 4-5 ROIs per FOV. At least 4-6 FOVs were taken from each organoid and 2 organoids per genotype were used for experiments. Measurements were taken using day 166-169 $Ctrl^2$ brain organoids. Error bars represent +/- S.E.M. Statistical significance (student's two-sided t-test) is represented by asterisks: $^{**}p < 0.01$. Source data and statistics are provided as a Source Data file.

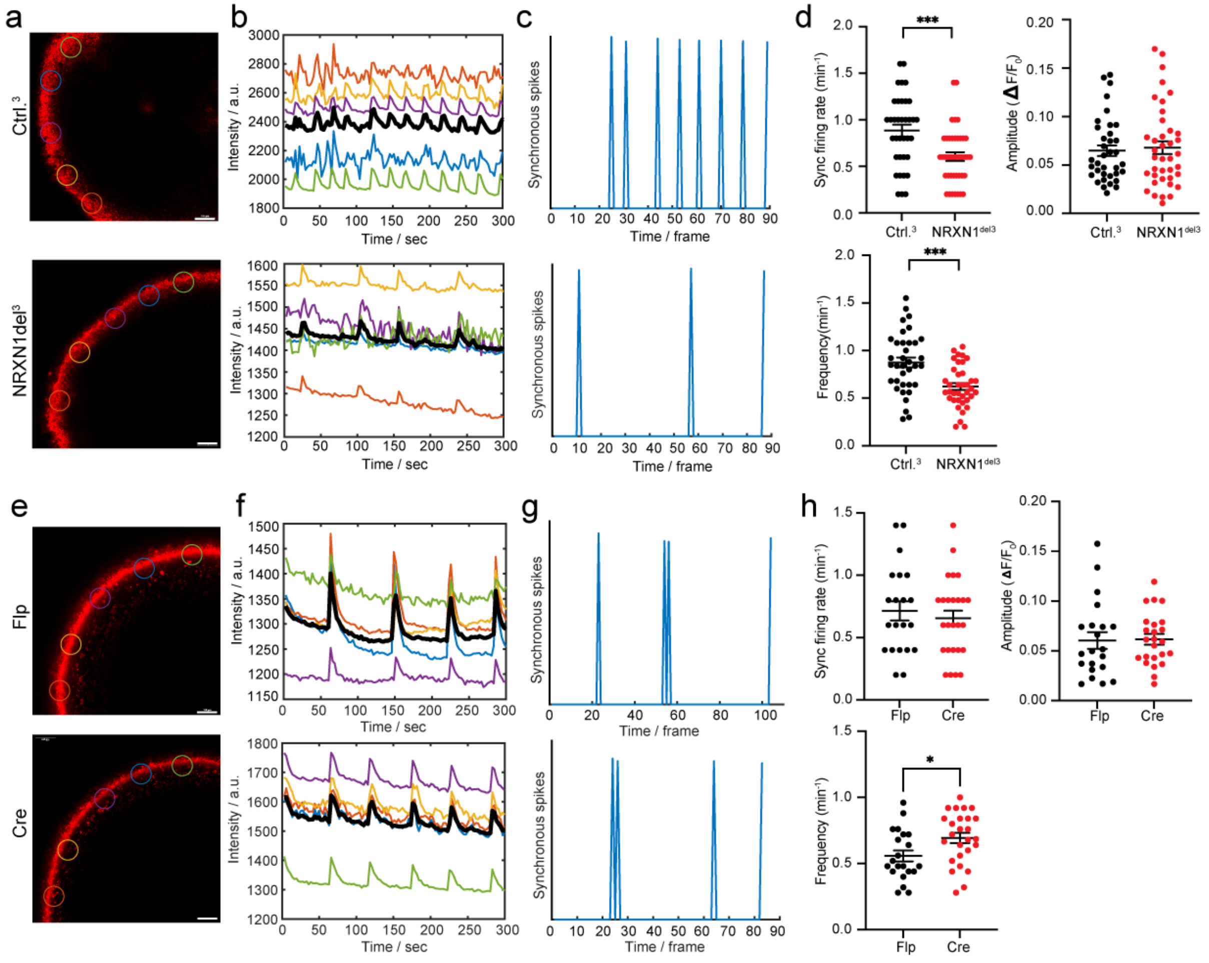

Figure S14. Impaired neuronal network activities in brain organoids carrying *NRXN1* deletions.

(a, e) Representative confocal images of brain organoids during live Ca^{2+} imaging (X-Rhod-1 dye) from intact engineered *NRXN1* cKO (e) and donor derived brain organoids (a; *NRXN1*^{del3} vs. Ctrl.^{3}) at days 130-160. Colored circles represent regions of interest (ROI) selected for analysis. (b, f) Corresponding colored raw intensity traces are shown in the boxed graphs with averaged intensities plotted in bolded black. Representative averaged synchronous spikes for each genotype are shown in c and g. (d, h) Averaged data for synchronous firing rates (number of detected synchronous spikes/minute) representative of network activity, as well as amplitudes (dF/F_0) and frequencies (total number of detected peaks/minute) of spontaneous spike activity, are shown in scatter plots. Each data point represents averaged data from a single field of view (FOV) consisting of 4-5 ROIs per FOV. At least 4-6 FOVs were taken from each organoid and 5-6 organoids per genotype were used for experiments. N=36 for Ctrl^{3}; N=38-41 for NRXN1^{del3}; N=21 for Flp; N=24-26 for Cre. Error bars represent +/- S.E.M. Student's t-test (two-sided) was performed for data shown for (d, h). Statistical significance is represented by asterisks: *p < 0.05, ***p < 0.001. Source data and statistics are provided as a Source Data file.

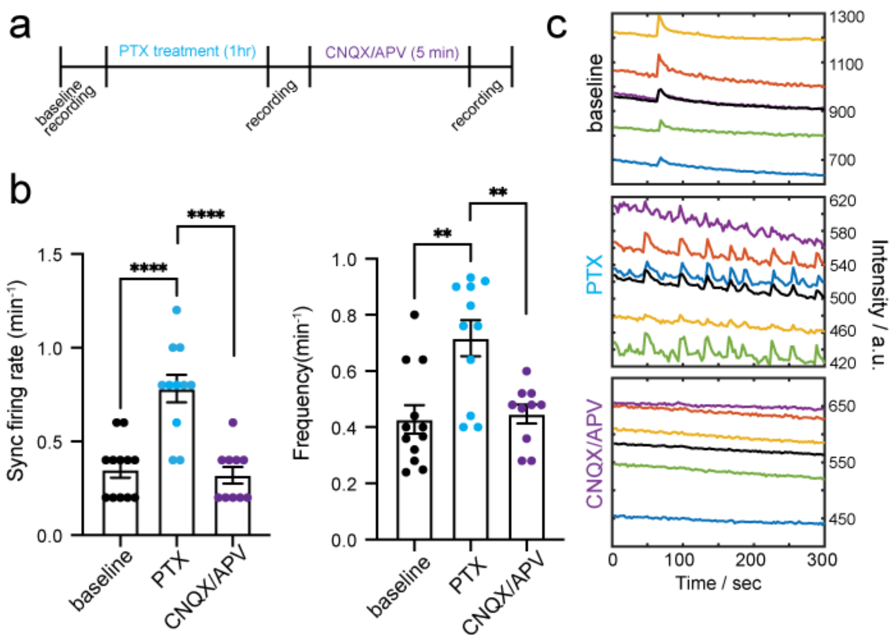

Figure S15. Calcium transients measured by X-Rhod-1 dye are sensitive to synaptic blockers.

(a) Schematic illustrating Ca^{2+} imaging workflow for PTX and CNQX/APV treatments in brain organoids infused with Ca^{2+} dye X-Rhod-1. (b) Average synchronous firing rate and neuronal spike frequency before (baseline) and after treatments (PTX, CNQX/APV). Treatment with GABAA receptor blocker PTX increases Ca^{2+} transient activity which is subsequently inhibited by CNQX/APV treatment, which blocks AMPARs and NMDARs respectively. N=10-12. (c) Representative traces showing raw intensity traces with averaged intensities plotted in bolded black for each condition. Each data point represents averaged data from a single field of view (FOV) consisting of 4-5 ROIs per FOV. At least 4-6 FOVs were taken from each organoid and 4 control organoids were used for experiments. Measurements were taken using day 109-115 Ctrl[2] brain organoids. Error bars represent +/- S.E.M. Statistical significance (student's two-sided t-test) is represented by asterisks: $**p < 0.01$ and $****p < 0.0001$. Source data and statistics are provided as a Source Data file.
