# Supplementary material for: Schizophrenia-associated NRXN1 deletions induce developmental-timing- and cell-type-specific vulnerabilities in human brain organoids
Source: Nat Commun. 2023 Jun 24;14:3770. doi: 10.1038/s41467-023-39420-6 (PMC10290702; doi:10.1038/s41467-023-39420-6)
Supplement: Supplementary file 2 — Description of Additional Supplementary Files [file 41467_2023_39420_MOESM2_ESM.pdf]

Description of Additional Supplementary Files

File Name: Supplementary Data 1
Description: Experimental inventory

File Name: Supplementary Data 2
Description: DEG table for NRXN1 cKO engineered organoids for all time points and cell types (filtered and unfiltered)

File Name: Supplementary Data 3
Description: DEG occurrence table for donor and engineered all time points and cell types

File Name: Supplementary Data 4
Description: GSEA for 3.5 mo NRXN1 cKO engineered organoids

File Name: Supplementary Data 5
Description: DEG table for SCZ-NRXN1^{del} donor derived organoids for all time points and cell types (filtered and unfiltered)

File Name: Supplementary Data 6
Description: GSEA for 3.5 mo overlapping DEGs in SCZ-NRXN1del derived organoids

File Name: Supplementary Data 7
Description: Neuronal splicing regulator DEGs at 3.5 mo

File Name: Supplementary Data 8
Description: Differential isoform expression in NRXN1 mutant iNs

File Name: Supplementary Data 9
Description: Differential local splicing in NRXN1 mutant iNs

File Name: Supplementary Data 10
Description: Overlap between DEGs and neuropsychiatric disorder GWAS loci
