# Supplementary material for: Schizophrenia-associated NRXN1 deletions induce developmental-timing- and cell-type-specific vulnerabilities in human brain organoids
Source: Nat Commun. 2023 Jun 24;14:3770. doi: 10.1038/s41467-023-39420-6 (PMC10290702; doi:10.1038/s41467-023-39420-6)
Supplement: Supplementary file 13 — Reporting Summary [file 41467_2023_39420_MOESM13_ESM.pdf]

Corresponding author(s): ChangHui Pak

Last updated by author(s): May 26, 2023

Reporting Summary

Nature Portfolio wishes to improve the reproducibility of the work that we publish. This form provides structure for consistency and transparency in reporting. For further information on Nature Portfolio policies, see our Editorial Policies and the Editorial Policy Checklist.

Statistics

For all statistical analyses, confirm that the following items are present in the figure legend, table legend, main text, or Methods section.

n/a	Confirmed	
☐	☒	The exact sample size (*n*) for each experimental group/condition, given as a discrete number and unit of measurement
☒	☐	A statement on whether measurements were taken from distinct samples or whether the same sample was measured repeatedly
☐	☒	The statistical test(s) used AND whether they are one- or two-sided<br>*Only common tests should be described solely by name; describe more complex techniques in the Methods section.*
☐	☒	A description of all covariates tested
☐	☒	A description of any assumptions or corrections, such as tests of normality and adjustment for multiple comparisons
☐	☒	A full description of the statistical parameters including central tendency (e.g. means) or other basic estimates (e.g. regression coefficient) AND variation (e.g. standard deviation) or associated estimates of uncertainty (e.g. confidence intervals)
☐	☒	For null hypothesis testing, the test statistic (e.g. *F*, *t*, *r*) with confidence intervals, effect sizes, degrees of freedom and *P* value noted<br>*Give P values as exact values whenever suitable.*
☒	☐	For Bayesian analysis, information on the choice of priors and Markov chain Monte Carlo settings
☒	☐	For hierarchical and complex designs, identification of the appropriate level for tests and full reporting of outcomes
☒	☐	Estimates of effect sizes (e.g. Cohen's *d*, Pearson's *r*), indicating how they were calculated

Our web collection on statistics for biologists contains articles on many of the points above.

Software and code

Policy information about availability of computer code

Data collection	Calcium imaging data and regular confocal imaging data were collected using Nikon's NIS-Elements imaging software (NIS-Elements Advance Research, Version 5).
Data analysis	Single cell data alignment was done by Cell Ranger 3.1.0 with reference genome GRCh38 (Ensembl 93). Single cell analysis scripts were written in Python (3.8.0) and R (4.0.2) and can be accessed in https://github.com/KANG-BIOINFO/NRXN1_effects (single cell data analysis). Seurat 4 and Scanpy 1.9.1 were used for single cell data preprocessing, integration, and analysis. Scrublet 0.1 was used to investigate doublets in data. ToppCell was used for gene module visualization. Monole3 (1.3.1) was used for pseudotime inference.<br>For bulk RNA-seq data, GRCh38.p13 was used as the reference genome and STAR 2.7.9 was used for alignment with gene annotation from Gencode v41. Genes and isoforms were quantified with RSEM 1.3.0. DESeq2 (1.41.1) was used for differential expression analysis. Majiq (2.3) was used to quantify alternative splicing.<br>Calcium imaging data analysis was done using MATLAB (Version, R2022b (9.13.0)). MATLAB scripts used for calcium imaging data analysis can be accessed in https://github.com/beccasbastian/CalciumIMG_Analysis. Statistical analysis of calcium imaging data was performed using Graphpad Prism version 9.5.1.

For manuscripts utilizing custom algorithms or software that are central to the research but not yet described in published literature, software must be made available to editors and reviewers. We strongly encourage code deposition in a community repository (e.g. GitHub). See the Nature Portfolio guidelines for submitting code & software for further information.

Data

Policy information about availability of data

All manuscripts must include a data availability statement. This statement should provide the following information, where applicable:

- Accession codes, unique identifiers, or web links for publicly available datasets
- A description of any restrictions on data availability
- For clinical datasets or third party data, please ensure that the statement adheres to our policy

Raw data from scRNA-seq can be accessed in the GEO repository via accession number: GSE228315 [https://www.ncbi.nlm.nih.gov/geo/query/acc.cgi?acc=GSE228315].

Human research participants

Policy information about studies involving human research participants and Sex and Gender in Research.

Reporting on sex and gender	N/A
Population characteristics	N/A
Recruitment	N/A
Ethics oversight	N/A

Note that full information on the approval of the study protocol must also be provided in the manuscript.

Field-specific reporting

Please select the one below that is the best fit for your research. If you are not sure, read the appropriate sections before making your selection.

☒ Life sciences ☐ Behavioural & social sciences ☐ Ecological, evolutionary & environmental sciences

For a reference copy of the document with all sections, see nature.com/documents/nr-reporting-summary-flat.pdf

Life sciences study design

All studies must disclose on these points even when the disclosure is negative.

Sample size

For scRNAseq, 3-5 organoids per genotype were pulled for single cell dissociation and used for sequencing for each time point. A total of 2 SCZ-NRXN1 del iPSC lines, 2 control donor iPSC lines, and 1 NRXN1 cKO isogenic line (Flp recombinase treated and Cre recombinase treated separately) were used for the study.

For immunohistochemistry, n=5 organoids per culture batch were collected at once and sectioned. At least 4 culture batches were grown independently. Random sections were used for staining and imaging.

For Ca2+ imaging, at least 4-7 organoids per genotype were used that came from > 3 independent culture batches. At least 4-6 field of views (FOV) per image per organoid was used.

No statistical methods were utilized to determine sample size. However, the lines utilized in this study is composed of rare variants, therefore we provide biological replicates (at least 2 replicate for each type of experiment) in addition to a conditional knock out line to support the findings in the study. Moreover, all these lines have been validated in previous publications (Pak et al., 2015 and Pak et al., 2021) to produce reliable phenotypes of the mutation.

Data exclusions

No data were excluded from analysis.

Replication

For scRNAseq, each line serves as a biological replicate which includes one replicate at 3 week, 2 month and 3.5 month for all SCZ-NRXN1 del iPSC lines and iPSC control lines. Additionally, for the NRXN1 cKO lines (FLP/CRE) 2 technical replicates were performed at 3.5 and 2 month while one replicate was performed for 3 weeks.
For immunohistochemistry, 3 independent batches were grown. Depending on need, additional 1-2 batches were grown and used for antibody testing.
For Ca2+ imaging, 3 independent culture batches were used for each genotype.
All attempts of replication was successful.

Randomization

Randomization is not relevant for this study as the cKO mutant (Cre) is paired with the isogenic control (Flp) from the same differentiation batch. For patient and control donor iPSC-derived organoids, we also cultured them altogether as paired entities across the same differentiation experiments over multiple batches.

Blinding

Investigators were blinded during Ca2+ imaging analysis. All transcriptomic analyses were done unblinded since all analyses were applied the same across all genotypes; no adjustments were made based of genotype.

Reporting for specific materials, systems and methods

We require information from authors about some types of materials, experimental systems and methods used in many studies. Here, indicate whether each material, system or method listed is relevant to your study. If you are not sure if a list item applies to your research, read the appropriate section before selecting a response.

Materials & experimental systems

n/a	Involved in the study
☐	☒ Antibodies
☐	☒ Eukaryotic cell lines
☒	☐ Palaeontology and archaeology
☒	☐ Animals and other organisms
☒	☐ Clinical data
☒	☐ Dual use research of concern

Methods

n/a	Involved in the study
☒	☐ ChIP-seq
☒	☐ Flow cytometry
☒	☐ MRI-based neuroimaging

Antibodies

Antibodies used

Primary antibodies used:
mouse anti-Ki67 (1:250, BD Biosciences BDB550609),
rabbit anti-SOX2 (1:500 Cell Signaling 3697S),
rabbit anti-HOPX (1:500, Proteintech 11419-1-H),
rat anti-CTIP2 (1:2000, Abcam ab18465),
rabbit anti-TBR2 (1:1000, Abcam ab23345),
mouse anti-SATB2 (1:1000 Abcam ab51502),
mouse anti-NEUN (1:500, EMD Millipore MAB377),
rabbit anti-NEUN (1:1000, EMD Milipore ABN78),
rabbit anti-S100B (1:1000, Sigma S2644),
chicken anti-MAP2 (1:5000, Abcam ab5392),
rabbit anti-SYNAPTOPHYSIN (1:1000, Abcam ab14692),
rabbit anti-HOMER (1:1000 Synaptic System 160003),
mouse anti-SYNAPSIN (1:500, Synaptic System 111011).

Secondary antibodies used:
All antibodies were diluted at 1:1000
Alexa Fluor goat anti-mouse 488, 546, 647 (Thermo Fisher Scientific A11001, A11003, A21236)
Alexa Fluor goat anti-rabbit 488, 546, 647 (Thermo Fisher Scientific A11034, A11010, A21245)
Alexa Fluor goat anti-chicken 488 (Thermo Fisher Scientific A11039)
Alexa Fluor goat anti-rat 647 (Thermo Fisher Scientific A21247)
DAPI (1:1000, Sigma MBD0015).

Validation

According to the manufacturer the mouse Ki-67 antibody is reactive to human and has been cited in 14 publications.
According to the manufacturer the rabbit SOX2 antibody is reactive to human and has been cited in 50 publications.
According to the manufacturer the rabbit HOPX antibody is reactive ti human and has been cited in 21 publications. This includes Badhuri et.al, 2020 which used this antibody on human forebrain organoids.
According to the manufacturer the rat CTIP2 antibody is reactive to human and has been cited 546 publications, which includes multiple human forebrain publications. Additionaly it has been validated in a protocol by Stem Cell Technologies (Cryogenic Tissue Processing and Section Immunofluorescence of Cerebral Organoids)
According to the manufacturer the rabbit TBR2 (EOMES) antibody is reactive to human and has been cited 403 publications. This includes Sloan et.al 2018 which used this antibody on human forebrain organoids.
According to the manufacturer the mouse SATB2 antibody is reactive to human and has been cited 206 publications. This includes Sloan et.al 2018 which used this antibody on human forebrain organoids.
According to the manufacturer the rabbit NEUN antibody is reactive to human and has been cited 4607 publications. This includes Sloan et.al 2018 which used this antibody on human forebrain organoids.
According to the manufacturer the rabbit S-100 antibody is reactive to human and has been cited in 86 publications.
According to the manufacturer the rabbit MAP2 antibody is reactive to human and has been cited 549 publications. Additionaly it has been validated in a protocol by Stem Cell Technologies (Cryogenic Tissue Processing and Section Immunofluorescence of Cerebral Organoids)
According to the manufacturer the rabbit SYNAPTOPHYSIN antibody is reactive to human and has been cited in 102 publications. This includes McSweeney et.al, 2022, a publication from our lab in induced human neurons.
According to the manufacturer the rabbit HOMER antibody is reactive to human and has been cited in 51 publications.
According to the manufacturer the rabbit SYNAPSIN antibody is reactive to human and has been cited in 51 publications.

Eukaryotic cell lines

Policy information about cell lines and Sex and Gender in Research

Cell line source(s)	NRXN1 cKO hESC line was provided by the Sudhof lab and was generated as outlined in Pak et al., 2015 (PMID 26279266). Control (C3141a, C9632, C8905a, C2320b) and SCZ-NRXN1 del iPSC lines (N3320a, N9540a, N1884a, N0575a) were generated from Pak et al., 2021 study (PMID 34035170). HEK293T was obtained from ATCC (catalog number: CRL-3216). The HEK293T has been validated in Pak et.al., 2021 for reliable generation of lentivirus.
Authentication	iPSC lines were authenticated by karyotyping and PCR genotyping. CNV analysis and WGS data are available for the iPSC lines through dbGAP (Pak et al., 2021 study).
Mycoplasma contamination	All cell lines were routinely tested for mycoplasma using Lookout Mycoplasma kit (Sigma). They were all negative.
Commonly misidentified lines (See ICLAC register)	No misidentified lines were used.
