# Supplementary figures and images for: Schizophrenia-associated NRXN1 deletions induce developmental-timing- and cell-type-specific vulnerabilities in human brain organoids
Source: Nat Commun. 2023 Jun 24;14:3770. doi: 10.1038/s41467-023-39420-6 (PMC10290702; doi:10.1038/s41467-023-39420-6)

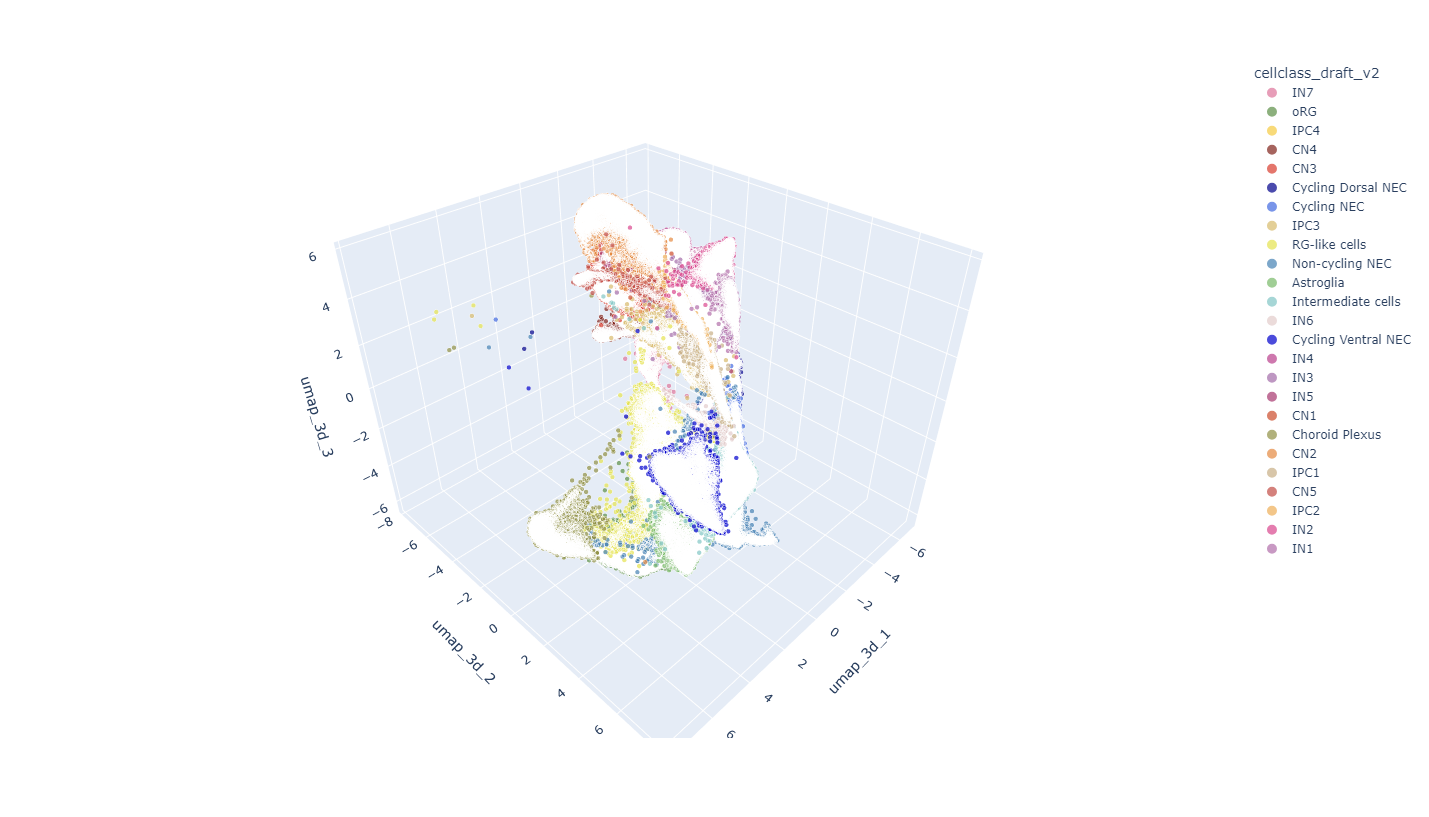

Supplement: Supplementary file 14 — Source Data [file 41467_2023_39420_MOESM14_ESM.zip › all_3d_v2.png]

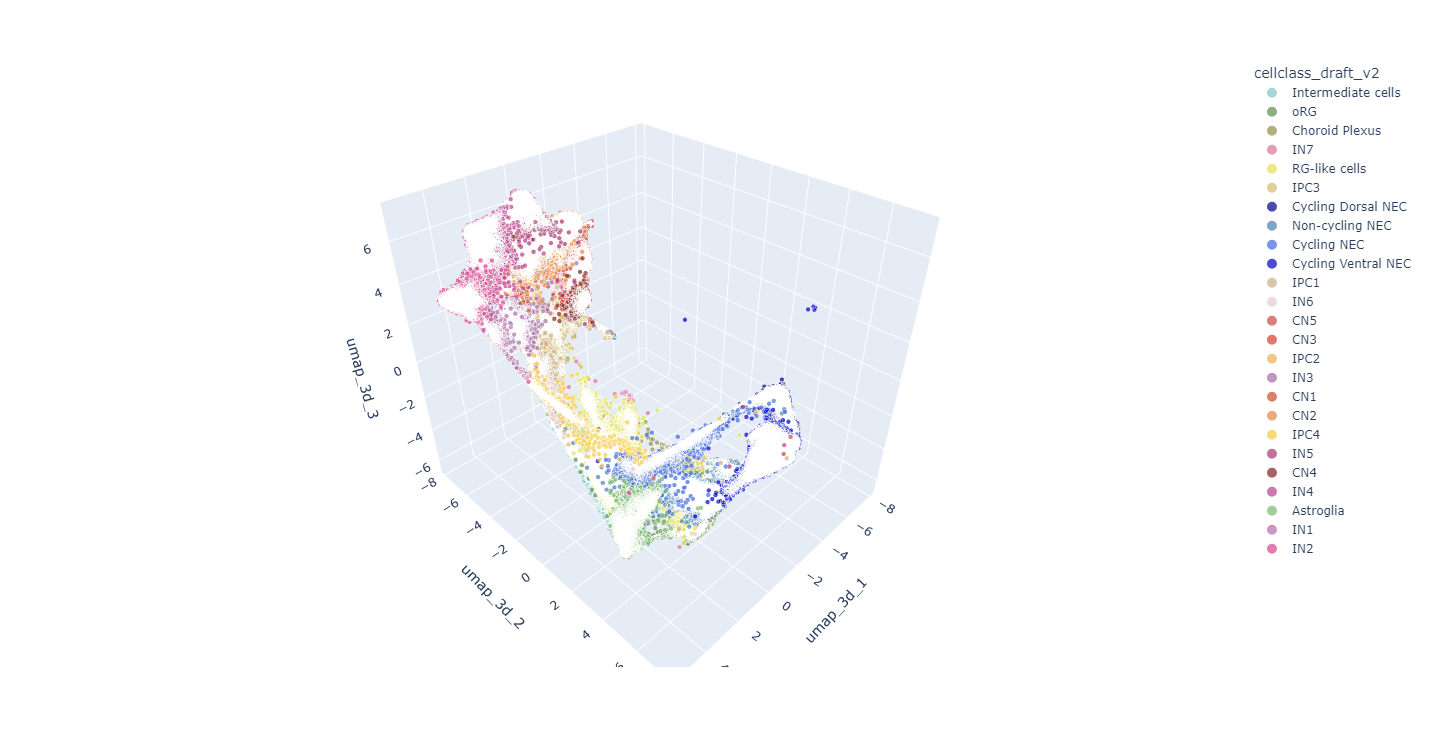

Supplement: Supplementary file 14 — Source Data [file 41467_2023_39420_MOESM14_ESM.zip › donor_3d_v2.png]

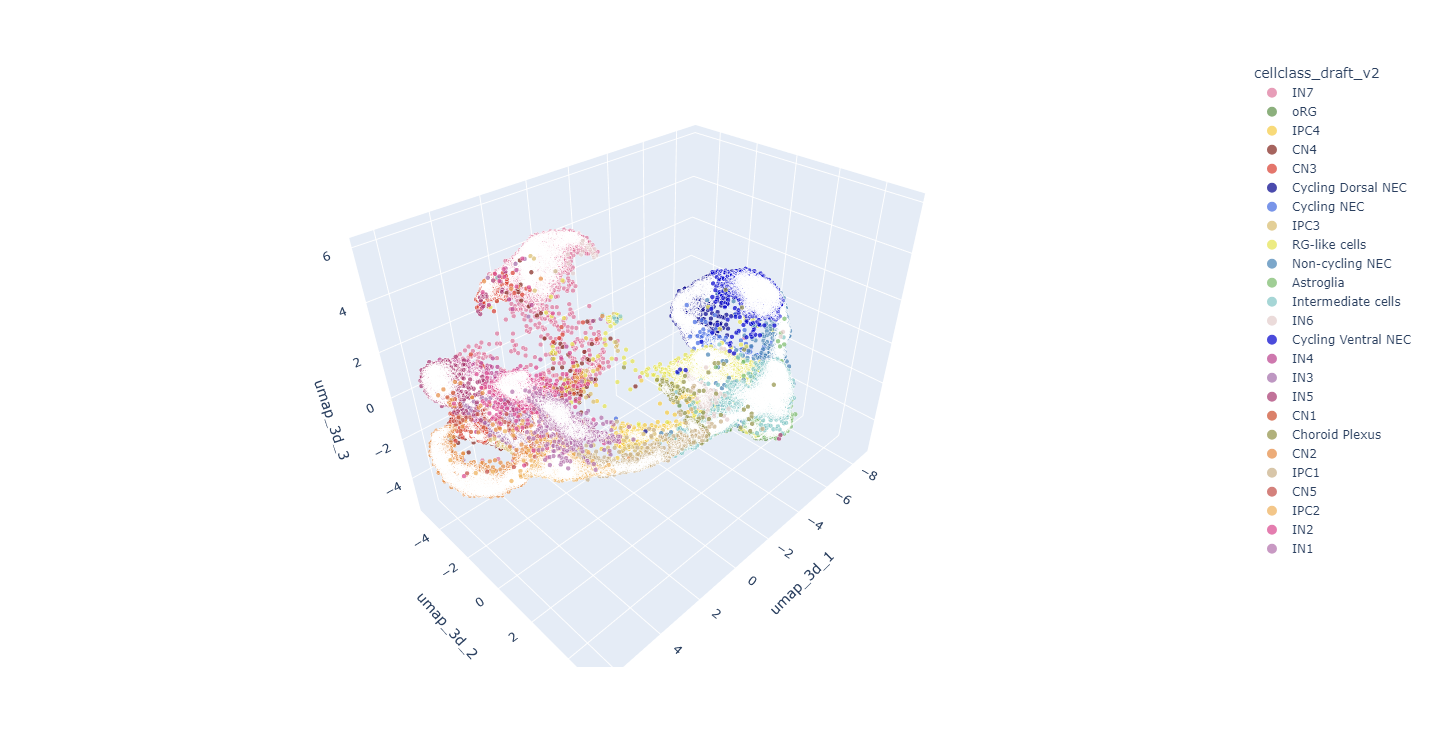

Supplement: Supplementary file 14 — Source Data [file 41467_2023_39420_MOESM14_ESM.zip › engineered_3d_v2.png]
